# Crystal structure of *trans*-(1,8-dibutyl-1,3,6,8,10,13-hexa­aza­cyclo­tetra­decane-κ^4^ *N* ^3^,*N* ^6^,*N* ^10^,*N* ^13^)bis­(perchlorato-κ*O*)copper(II) from synchrotron data

**DOI:** 10.1107/S2056989014028047

**Published:** 2015-01-10

**Authors:** Dae-Woong Kim, Jong Won Shin, Dohyun Moon

**Affiliations:** a Beamline Department, Pohang Accelerator Laboratory/POSTECH 80, Pohang 790-784, South Korea

**Keywords:** Crystal structure, aza­macrocyclic ligand, Jahn–Teller distortion, synchrotron data, hydrogen bonds

## Abstract

The structure of the title compound, [Cu(ClO_4_)_2_(C_16_H_38_N_6_)] has been determined from synchrotron data, λ = 0.62988 Å. The asymmetric unit comprises one half of the Cu^II^ complex as the Cu^II^ cation lies on an inversion center. It is coordinated by the four secondary N atoms of the macrocyclic ligand and the mutually *trans* O atoms of the two perchlorate ions in a tetra­gonally distorted octa­hedral geometry. The average equatorial Cu—N bond length is significantly shorter than the average axial Cu—O bond length [2.010 (4) and 2.569 (1) Å, respectively]. Intra­molecular N—H⋯O hydrogen bonds between the macrocyclic ligand and uncoordinating O atoms of the perchlorate ligand stabilize the mol­ecular structure. In the crystal structure, an extensive series of inter­molecular N—H⋯O and C—H⋯O hydrogen bonds generate a three-dimensional network.

## Abstract 2

The Cu^II^ ion in the title compound shows a slightly distorted octa­hedral coordination geometry with four N atoms of the aza­macrocyclic ligand and two perchlorate anions. In the crystal, mol­ecules are linked by N–H⋯O and C–H⋯O hydrogen bonds, forming a three-dimensional network.

## Chemical context   

Coordination compounds with macrocyclic ligands have attracted considerable attention in chemistry, biological chemistry and materials science (Lehn, 1995 ▸). In particular, macrocyclic Cu^II^ complexes with vacant sites in the axial positions are good building blocks for assembling multi-dimensional frameworks (Ko *et al.*, 2002 ▸), with potential applications as metal extractants, radiotherapeutic materials and as medical imaging agents (Sowen *et al.*, 2013 ▸). For example, Cu^II^ complexes with tetra-aza­macrocyclic ligands have been studied with various auxiliary anionic ligands such as ferricyanide and hexacyanidochromate and their biological redox-sensing and magnetic properties (Xiang *et al.*, 2009 ▸) have been investigated. Moreover, the perchlorate ion is a versatile anion which can easily bridge two transition metal complexes, allowing the assembly of multi-dimensional compounds (Kwak *et al.*, 2001 ▸).
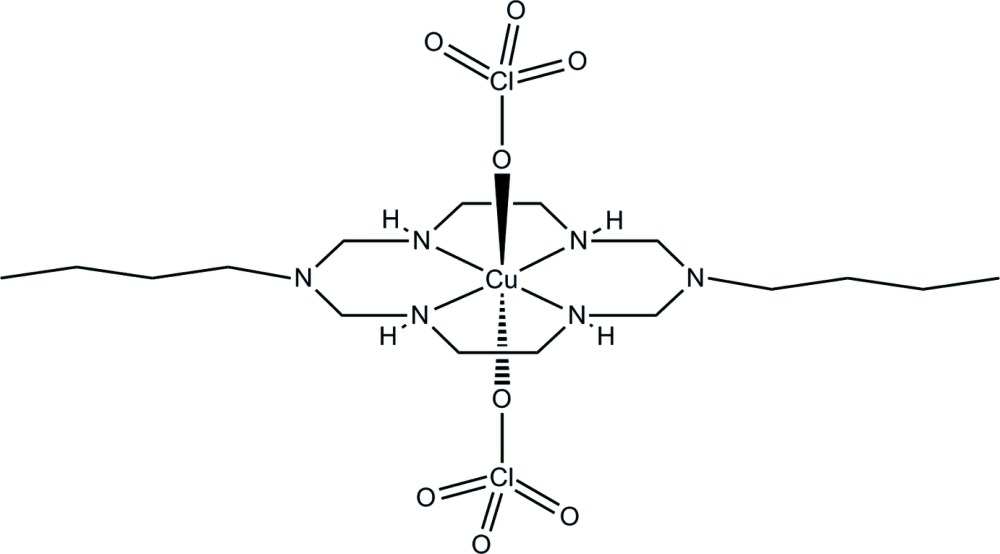


Here, we report the synthesis and crystal structure of a Cu^II^ aza­macrocyclic complex, *trans*-(1,8-dibutyl-1,3,6,8,10,13-hexaaza­cyclo­tetra­decane-κ^4^ *N* ^3^,*N* ^6^,*N* ^10^,*N* ^13^)bis­(perchlorato-κ*O*)copper(II), which has two perchlorate ions coordinating in the axial positions of the overall six-coordinate complex.

## Structural commentary   

**Figure 1:**
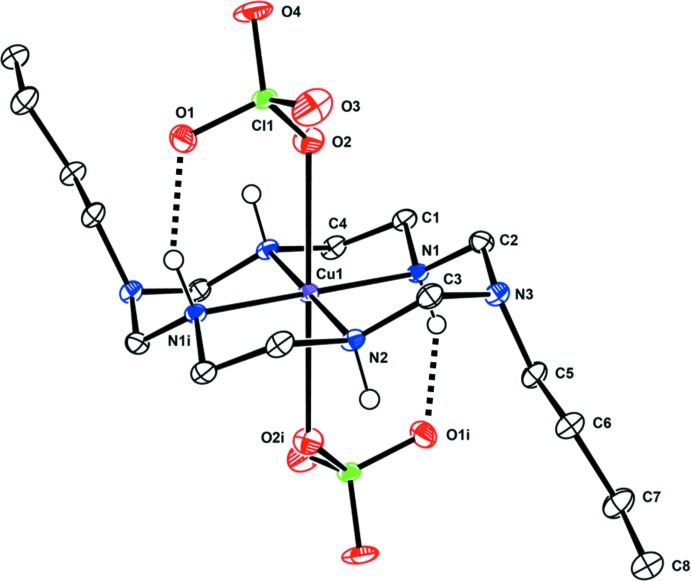
View of the mol­ecular structure of the title compound, showing the atom labelling scheme, with displacement ellipsoids drawn at the 50% probability level. H atoms bonded to C atoms have been omitted for clarity. Intra­molecular N—H⋯O hydrogen bonds are shown as black dashed lines. [Symmetry code: (i) −*x* + 1, −*y* + 1, −*z* + 1.]

In the title compound, the coordination environment around the Cu^II^ ion, which lies on an inversion center, is tetra­gonally distorted octa­hedral. The copper(II) ion binds to the four secondary N atoms of the aza­macrocyclic ligand in a square-planar fashion in the equatorial plane, with two O atoms from the perchlorate anions in axial positions as shown in Fig. 1 ▸. The bonds to the two axially located perchlorate anions are significantly longer than those to the donor N atoms in the equatorial plane. This can be attributed either to a rather large Jahn–Teller distortion of the Cu^II^ ion and/or to a considerable ring contraction of the aza­macrocyclic ligand (Halcrow, 2013 ▸). The six-membered chelate rings adopt chair conformations and the five-membered chelate rings assume *gauche* conformations (Min & Suh, 2001 ▸). Intra­molecular N—H⋯O hydrogen bonds between the secondary amine groups of the aza­macrocyclic ligand and an O atom of each perchlorate ion contribute to the mol­ecular conformation (Fig. 1 ▸ and Table 1 ▸).

## Supra­molecular features   

**Figure 2:**
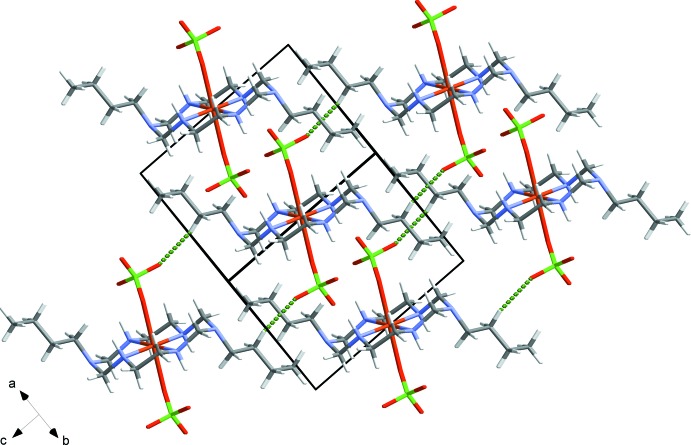
View of the contacts made by an individual complex mol­ecule with hydrogen bonds drawn as dashed lines.

**Figure 3:**
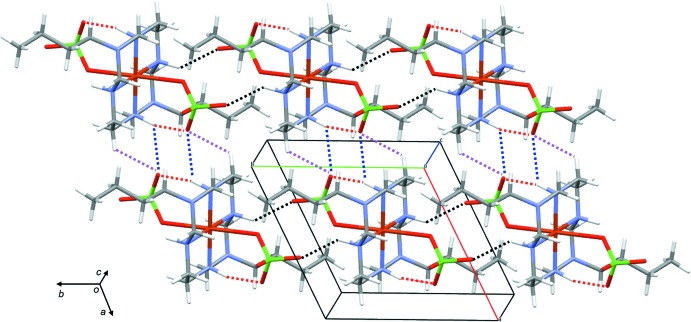
Sheets of complex mol­ecules in the *ab* plane. Hydrogen-bonding interactions are shown as dashed lines.

Each complex mol­ecule forms three N—H⋯O and two C—H⋯O hydrogen bonds (Steed & Atwood, 2009 ▸), as shown in Table 1 ▸, Fig. 2 ▸. Sheets of complex mol­ecules form in the *ab* plane, Fig. 3 ▸, and additional C6—H6B⋯O3 contacts link these sheets into a three-dimensional network.

## Database survey   

A search of the Cambridge Structural Database (Version 5.35, May 2014 with three updates; Groom & Allen 2014 ▸) indicated that 51 aza­macrocyclic Cu^II^ complexes with pendant alkyl groups had been reported previously. These complexes have been studied as good building blocks for supra­molecular chemistry and contain a variety of pendant alkyl groups (Cho *et al.*, 2003 ▸). Their magnetic properties and guest-exchange effects with cyanido groups and carb­oxy­lic acid groups as ligands have also been investigated (Ko *et al.*, 2002 ▸; Zhou *et al.*, 2014 ▸). No corresponding aza­macrocyclic Cu^II^ complex with pendant butyl groups has been reported and the title compound was newly synthesized for this research.

## Synthesis and crystallization   

The title compound was prepared as follows. Ethyl­enedi­amine (3.4 mL, 0.05 mol), paraformaldehyde (3.0 g, 0.10 mol), and butyl­amine (3.7 g, 0.05 mol) were slowly added to a stirred solution of CuCl_2_·2H_2_O (4.26 g, 0.025 mol) in MeOH (50 mL). The mixture was heated to reflux for 1 day. The solution was filtered and cooled at room temperature. HClO_4_ (70%, 15 mL) was added to the purple solution. A bright-purple precipitate formed and was filtered off, washed with H_2_O, MeOH, and diethyl ether, and dried in air. Purple crystals of the title compound were obtained by diffusion of diethyl ether into the purple solution over several days. Yield: 2.38g (17%). FT–IR (ATR, cm^−1^): 3240, 2936, 1443, 1053, 995, 962, 746. 

**Safety note**: Although we have experienced no problems with the compound reported in this study, perchlorate salts of metal complexes are often explosive and should be handled with great caution.

## Refinement   

Crystal data, data collection and structure refinement details are summarized in Table 2 ▸. All H atoms were placed in geometrically idealized positions and constrained to ride on their parent atoms, with C—H distances of 0.98–0.99 Å and an N—H distance of 1.0 Å with *U*_iso_(H) values of 1.2 or 1.5 *U*_eq_ of the parent atoms.

## Supplementary Material

Crystal structure: contains datablock(s) I. DOI: 10.1107/S2056989014028047/sj5435sup1.cif

Structure factors: contains datablock(s) I. DOI: 10.1107/S2056989014028047/sj5435Isup2.hkl

CCDC reference: 1040897

Additional supporting information:  crystallographic information; 3D view; checkCIF report

## Figures and Tables

**Table 1:** Hydrogen-bond geometry (, )

*D*H*A*	*D*H	H*A*	*D* *A*	*D*H*A*
N1H1O1^i^	1.00	2.50	3.136(2)	121
N2H2O4^ii^	1.00	2.17	3.000(2)	139
C1H1AO1^i^	0.99	2.46	3.160(2)	127
N1H1O1^iii^	1.00	2.08	3.018(2)	155
C6H6BO3^iv^	0.99	2.50	3.338(3)	142

Symmetry codes: (i) 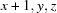; (ii) 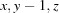; (iii) 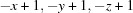; (iv) 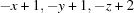.

**Table 2:** Experimental details

Crystal data	
Chemical formula	[Cu(ClO_4_)_2_(C_16_H_38_N_6_)]
*M*_r_	576.96
Crystal system, space group	Triclinic, *P* 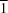
Temperature (K)	100
*a*, *b*, *c* ()	8.2230(16), 8.3600(17), 10.039(2)
, , ()	92.87(3), 96.12(3), 116.60(3)
*V* (^3^)	609.8(3)
*Z*	1
Radiation type	Synchrotron, = 0.62998
(mm^1^)	0.84
Crystal size (mm)	0.10 0.10 0.03

Data collection	
Diffractometer	ADSC Q210 CCD area detector
Absorption correction	Empirical (using intensity measurements) *HKL3000sm* *SCALEPACK* (Otwinowski Minor, 1997 ▸)
*T*_min_, *T*_max_	0.921, 0.975
No. of measured, independent and observed [*I* > 2(*I*)] reflections	6292, 3195, 2536
*R*_int_	0.025
(sin /)_max_ (^1^)	0.696

Refinement	
*R*[*F* ^2^ > 2(*F* ^2^)], *wR*(*F* ^2^), *S*	0.034, 0.091, 1.02
No. of reflections	3195
No. of parameters	152
H-atom treatment	H-atom parameters constrained
_max_, _min_ (e ^3^)	0.29, 0.86

Computer programs: *PAL ADSC Quantum-210 ADX* (Arvai Nielsen, 1983 ▸), *HKL3000sm* (Otwinowski Minor, 1997 ▸), *SHELXT2014/4* and *SHELXL2014*/7 (Sheldrick, 2008 ▸), *ORTEP-3 for Windows* (Farrugia, 2012 ▸)and *publCIF* (Westrip, 2010 ▸).

## supplementary crystallographic information

### Crystal data

**Table:**

[Cu(ClO_4_)_2_(C_16_H_38_N_6_)]	*Z* = 1
*M**_r_* = 576.96	*F*(000) = 303
Triclinic, *P*1	*D*_x_ = 1.571 Mg m^−^^3^
*a* = 8.2230 (16) Å	Synchrotron radiation, λ = 0.62998 Å
*b* = 8.3600 (17) Å	Cell parameters from 16838 reflections
*c* = 10.039 (2) Å	θ = 0.4–33.6°
α = 92.87 (3)°	µ = 0.84 mm^−^^1^
β = 96.12 (3)°	*T* = 100 K
γ = 116.60 (3)°	Plate, purple
*V* = 609.8 (3) Å^3^	0.10 × 0.10 × 0.03 mm

### Data collection

**Table:**

ADSC Q210 CCD area-detector diffractometer	2536 reflections with *I* > 2σ(*I*)
Radiation source: PLSII 2D bending magnet	*R*_int_ = 0.025
ω scans	θ_max_ = 26.0°, θ_min_ = 1.8°
Absorption correction: empirical (using intensity measurements) (*HKL3000sm**SCALEPACK*; Otwinowski & Minor, 1997)	*h* = −11→11
*T*_min_ = 0.921, *T*_max_ = 0.975	*k* = −11→11
6292 measured reflections	*l* = −13→13
3195 independent reflections	

### Refinement

**Table:**

Refinement on *F*^2^	0 restraints
Least-squares matrix: full	Hydrogen site location: inferred from neighbouring sites
*R*[*F*^2^ > 2σ(*F*^2^)] = 0.034	H-atom parameters constrained
*wR*(*F*^2^) = 0.091	*w* = 1/[σ^2^(*F*_o_^2^) + (0.0574*P*)^2^] where *P* = (*F*_o_^2^ + 2*F*_c_^2^)/3
*S* = 1.02	(Δ/σ)_max_ = 0.001
3195 reflections	Δρ_max_ = 0.29 e Å^−^^3^
152 parameters	Δρ_min_ = −0.86 e Å^−^^3^

### Special details

**Table:**

Geometry. All e.s.d.'s (except the e.s.d. in the dihedral angle between two l.s. planes) are estimated using the full covariance matrix. The cell e.s.d.'s are taken into account individually in the estimation of e.s.d.'s in distances, angles and torsion angles; correlations between e.s.d.'s in cell parameters are only used when they are defined by crystal symmetry. An approximate (isotropic) treatment of cell e.s.d.'s is used for estimating e.s.d.'s involving l.s. planes.

### Fractional atomic coordinates and isotropic or equivalent isotropic displacement parameters (Å^2^)

**Table:**

	*x*	*y*	*z*	*U*_iso_*/*U*_eq_	
Cu1	0.5000	0.5000	0.5000	0.01063 (10)	
N1	0.7678 (2)	0.6261 (2)	0.57656 (16)	0.0119 (3)	
H1	0.8221	0.5443	0.5535	0.014*	
N2	0.4291 (2)	0.3507 (2)	0.65492 (16)	0.0123 (3)	
H2	0.4599	0.2490	0.6389	0.015*	
N3	0.7247 (2)	0.5222 (2)	0.80108 (16)	0.0152 (3)	
C1	0.8525 (2)	0.7860 (2)	0.5034 (2)	0.0155 (4)	
H1A	0.9876	0.8328	0.5156	0.019*	
H1B	0.8242	0.8821	0.5385	0.019*	
C2	0.8084 (3)	0.6731 (3)	0.7267 (2)	0.0164 (4)	
H2A	0.9432	0.7303	0.7541	0.020*	
H2B	0.7654	0.7626	0.7508	0.020*	
C3	0.5278 (3)	0.4466 (3)	0.7910 (2)	0.0158 (4)	
H3A	0.4948	0.5445	0.8119	0.019*	
H3B	0.4855	0.3612	0.8594	0.019*	
C4	0.2256 (2)	0.2705 (3)	0.6448 (2)	0.0156 (4)	
H4A	0.1894	0.3598	0.6819	0.019*	
H4B	0.1786	0.1649	0.6964	0.019*	
C5	0.8000 (3)	0.3919 (3)	0.7910 (2)	0.0169 (4)	
H5A	0.9359	0.4586	0.8041	0.020*	
H5B	0.7593	0.3265	0.6992	0.020*	
C6	0.7409 (3)	0.2567 (3)	0.8931 (2)	0.0177 (4)	
H6A	0.6067	0.1782	0.8721	0.021*	
H6B	0.7662	0.3217	0.9840	0.021*	
C7	0.8401 (3)	0.1406 (3)	0.8937 (2)	0.0221 (4)	
H7A	0.9723	0.2171	0.9266	0.026*	
H7B	0.8289	0.0888	0.8003	0.026*	
C8	0.7636 (3)	−0.0116 (3)	0.9822 (2)	0.0215 (4)	
H8A	0.7749	0.0390	1.0749	0.032*	
H8B	0.8328	−0.0812	0.9806	0.032*	
H8C	0.6338	−0.0904	0.9481	0.032*	
Cl1	0.32457 (6)	0.78356 (6)	0.65025 (4)	0.01406 (11)	
O1	0.1610 (2)	0.6610 (2)	0.56090 (18)	0.0282 (4)	
O2	0.48300 (19)	0.77847 (19)	0.60352 (16)	0.0221 (3)	
O3	0.3102 (3)	0.7249 (2)	0.78200 (16)	0.0319 (4)	
O4	0.3413 (2)	0.96148 (19)	0.65392 (18)	0.0285 (4)	

### Atomic displacement parameters (Å^2^)

**Table:**

	*U*^11^	*U*^22^	*U*^33^	*U*^12^	*U*^13^	*U*^23^
Cu1	0.00685 (15)	0.00798 (15)	0.01599 (17)	0.00179 (11)	0.00383 (11)	0.00327 (12)
N1	0.0092 (7)	0.0080 (6)	0.0180 (8)	0.0032 (5)	0.0030 (5)	0.0031 (6)
N2	0.0091 (6)	0.0114 (7)	0.0184 (8)	0.0053 (5)	0.0054 (6)	0.0044 (6)
N3	0.0152 (7)	0.0159 (8)	0.0170 (8)	0.0091 (6)	0.0028 (6)	0.0022 (6)
C1	0.0082 (8)	0.0091 (8)	0.0275 (10)	0.0015 (6)	0.0051 (7)	0.0069 (7)
C2	0.0143 (8)	0.0125 (8)	0.0208 (10)	0.0052 (7)	0.0009 (7)	0.0000 (7)
C3	0.0156 (9)	0.0176 (9)	0.0176 (9)	0.0100 (7)	0.0050 (7)	0.0034 (8)
C4	0.0094 (8)	0.0141 (8)	0.0253 (10)	0.0047 (7)	0.0096 (7)	0.0101 (8)
C5	0.0148 (8)	0.0199 (9)	0.0202 (9)	0.0112 (7)	0.0039 (7)	0.0046 (8)
C6	0.0197 (9)	0.0191 (9)	0.0194 (10)	0.0124 (8)	0.0057 (7)	0.0059 (8)
C7	0.0186 (9)	0.0242 (10)	0.0295 (11)	0.0133 (8)	0.0081 (8)	0.0100 (9)
C8	0.0242 (10)	0.0202 (10)	0.0244 (11)	0.0133 (8)	0.0050 (8)	0.0054 (8)
Cl1	0.0128 (2)	0.0121 (2)	0.0198 (2)	0.00762 (17)	0.00396 (16)	0.00067 (18)
O1	0.0152 (7)	0.0258 (8)	0.0420 (10)	0.0115 (6)	−0.0050 (6)	−0.0127 (7)
O2	0.0132 (6)	0.0216 (7)	0.0332 (8)	0.0088 (6)	0.0086 (6)	−0.0005 (6)
O3	0.0450 (10)	0.0412 (10)	0.0225 (8)	0.0280 (8)	0.0144 (7)	0.0124 (8)
O4	0.0297 (8)	0.0123 (7)	0.0490 (11)	0.0132 (6)	0.0101 (7)	0.0047 (7)

### Geometric parameters (Å, º)

**Table:**

Cu1—N1	2.0073 (17)	C4—C1^i^	1.518 (3)
Cu1—N1^i^	2.0073 (17)	C4—H4A	0.9900
Cu1—N2^i^	2.0131 (17)	C4—H4B	0.9900
Cu1—N2	2.0131 (17)	C5—C6	1.515 (3)
N1—C1	1.478 (2)	C5—H5A	0.9900
N1—C2	1.501 (3)	C5—H5B	0.9900
N1—H1	1.0000	C6—C7	1.522 (3)
N2—C4	1.487 (2)	C6—H6A	0.9900
N2—C3	1.496 (3)	C6—H6B	0.9900
N2—H2	1.0000	C7—C8	1.523 (3)
N3—C2	1.432 (2)	C7—H7A	0.9900
N3—C3	1.440 (2)	C7—H7B	0.9900
N3—C5	1.478 (2)	C8—H8A	0.9800
C1—C4^i^	1.518 (3)	C8—H8B	0.9800
C1—H1A	0.9900	C8—H8C	0.9800
C1—H1B	0.9900	Cl1—O4	1.4293 (15)
C2—H2A	0.9900	Cl1—O3	1.4318 (17)
C2—H2B	0.9900	Cl1—O1	1.4420 (17)
C3—H3A	0.9900	Cl1—O2	1.4481 (14)
C3—H3B	0.9900		
			
N1—Cu1—N1^i^	180.00 (9)	H3A—C3—H3B	107.7
N1—Cu1—N2^i^	86.45 (7)	N2—C4—C1^i^	107.28 (15)
N1^i^—Cu1—N2^i^	93.55 (7)	N2—C4—H4A	110.3
N1—Cu1—N2	93.55 (7)	C1^i^—C4—H4A	110.3
N1^i^—Cu1—N2	86.45 (7)	N2—C4—H4B	110.3
N2^i^—Cu1—N2	180.0	C1^i^—C4—H4B	110.3
C1—N1—C2	112.37 (15)	H4A—C4—H4B	108.5
C1—N1—Cu1	106.33 (11)	N3—C5—C6	113.14 (15)
C2—N1—Cu1	115.28 (12)	N3—C5—H5A	109.0
C1—N1—H1	107.5	C6—C5—H5A	109.0
C2—N1—H1	107.5	N3—C5—H5B	109.0
Cu1—N1—H1	107.5	C6—C5—H5B	109.0
C4—N2—C3	113.49 (15)	H5A—C5—H5B	107.8
C4—N2—Cu1	106.73 (11)	C5—C6—C7	112.19 (16)
C3—N2—Cu1	115.29 (12)	C5—C6—H6A	109.2
C4—N2—H2	107.0	C7—C6—H6A	109.2
C3—N2—H2	107.0	C5—C6—H6B	109.2
Cu1—N2—H2	107.0	C7—C6—H6B	109.2
C2—N3—C3	114.81 (15)	H6A—C6—H6B	107.9
C2—N3—C5	114.08 (15)	C6—C7—C8	112.45 (16)
C3—N3—C5	116.15 (16)	C6—C7—H7A	109.1
N1—C1—C4^i^	107.87 (15)	C8—C7—H7A	109.1
N1—C1—H1A	110.1	C6—C7—H7B	109.1
C4^i^—C1—H1A	110.1	C8—C7—H7B	109.1
N1—C1—H1B	110.1	H7A—C7—H7B	107.8
C4^i^—C1—H1B	110.1	C7—C8—H8A	109.5
H1A—C1—H1B	108.4	C7—C8—H8B	109.5
N3—C2—N1	114.03 (15)	H8A—C8—H8B	109.5
N3—C2—H2A	108.7	C7—C8—H8C	109.5
N1—C2—H2A	108.7	H8A—C8—H8C	109.5
N3—C2—H2B	108.7	H8B—C8—H8C	109.5
N1—C2—H2B	108.7	O4—Cl1—O3	110.11 (11)
H2A—C2—H2B	107.6	O4—Cl1—O1	109.74 (10)
N3—C3—N2	113.39 (15)	O3—Cl1—O1	108.36 (11)
N3—C3—H3A	108.9	O4—Cl1—O2	110.65 (10)
N2—C3—H3A	108.9	O3—Cl1—O2	108.82 (10)
N3—C3—H3B	108.9	O1—Cl1—O2	109.12 (9)
N2—C3—H3B	108.9		
			
C2—N1—C1—C4^i^	−169.53 (14)	C4—N2—C3—N3	179.24 (14)
Cu1—N1—C1—C4^i^	−42.53 (15)	Cu1—N2—C3—N3	−57.23 (18)
C3—N3—C2—N1	−69.8 (2)	C3—N2—C4—C1^i^	168.24 (15)
C5—N3—C2—N1	67.8 (2)	Cu1—N2—C4—C1^i^	40.14 (16)
C1—N1—C2—N3	178.48 (14)	C2—N3—C5—C6	167.34 (16)
Cu1—N1—C2—N3	56.43 (18)	C3—N3—C5—C6	−55.6 (2)
C2—N3—C3—N2	70.2 (2)	N3—C5—C6—C7	−172.24 (17)
C5—N3—C3—N2	−66.5 (2)	C5—C6—C7—C8	−172.38 (18)

Symmetry code: (i) −*x*+1, −*y*+1, −*z*+1.

### Hydrogen-bond geometry (Å, º)

**Table:**

*D*—H···*A*	*D*—H	H···*A*	*D*···*A*	*D*—H···*A*
N1—H1···O1^ii^	1.00	2.50	3.136 (2)	121
N2—H2···O4^iii^	1.00	2.17	3.000 (2)	139
C1—H1*A*···O1^ii^	0.99	2.46	3.160 (2)	127
N1—H1···O1^i^	1.00	2.08	3.018 (2)	155
C6—H6*B*···O3^iv^	0.99	2.50	3.338 (3)	142

Symmetry codes: (i) −*x*+1, −*y*+1, −*z*+1; (ii) *x*+1, *y*, *z*; (iii) *x*, *y*−1, *z*; (iv) −*x*+1, −*y*+1, −*z*+2.
